# Ribosome Profiling and Mass Spectrometry Reveal Widespread Mitochondrial Translation Defects in a Striatal Cell Model of Huntington Disease

**DOI:** 10.1016/j.mcpro.2024.100746

**Published:** 2024-03-05

**Authors:** Sunayana Dagar, Manish Sharma, George Tsaprailis, Catherina Scharager Tapia, Gogce Crynen, Preksha Sandipkumar Joshi, Neelam Shahani, Srinivasa Subramaniam

**Affiliations:** 1 Department of Neuroscience, The Herbert Wertheim UF Scripps Institute for Biomedical Innovation & Technology, Jupiter, Florida, USA; 2 Proteomics Core, The Wertheim UF Scripps Institute, Jupiter, Florida, USA; 3 Bioinformatics and Statistics Core, The Wertheim UF Scripps Institute, Jupiter, Florida, USA; 4 The Skaggs Graduate School of Chemical and Biological Sciences, The Scripps Research Institute, La Jolla, California, USA; 5 Norman Fixel Institute for Neurological Diseases, Gainesville, Florida, USA

**Keywords:** dichotomy, energy metabolism, abnormality, brain disease, vulnerability, oxidative stress, mitoribosome, cytoribosome, mRNA translation

## Abstract

Huntington disease (HD) is caused by an expanded polyglutamine mutation in huntingtin (mHTT) that promotes prominent atrophy in the striatum and subsequent psychiatric, cognitive deficits, and choreiform movements. Multiple lines of evidence point to an association between HD and aberrant striatal mitochondrial functions; however, the present knowledge about whether (or how) mitochondrial mRNA translation is differentially regulated in HD remains unclear. We found that protein synthesis is diminished in HD mitochondria compared to healthy control striatal cell models. We utilized ribosome profiling (Ribo-Seq) to analyze detailed snapshots of ribosome occupancy of the mitochondrial mRNA transcripts in control and HD striatal cell models. The Ribo-Seq data revealed almost unaltered ribosome occupancy on the nuclear-encoded mitochondrial transcripts involved in oxidative phosphorylation (SDHA, Ndufv1, Timm23, Tomm5, Mrps22) in HD cells. By contrast, ribosome occupancy was dramatically increased for mitochondrially encoded oxidative phosphorylation mRNAs (mt-Nd1, mt-Nd2, mt-Nd4, mt-Nd4l, mt-Nd5, mt-Nd6, mt-Co1, mt-Cytb, and mt-ATP8). We also applied tandem mass tag–based mass spectrometry identification of mitochondrial proteins to derive correlations between ribosome occupancy and actual mature mitochondrial protein products. We found many mitochondrial transcripts with comparable or higher ribosome occupancy, but diminished mitochondrial protein products, in HD. Thus, our study provides the first evidence of a widespread dichotomous effect on ribosome occupancy and protein abundance of mitochondria-related genes in HD.

## Highlights

• Decreased mitochondrial protein synthesis in HD.
• Enhanced ribosome occupancy in mitochondria-encoded mRNAs in HD.
• TMT-based proteomics showed significant mitochondrial proteome changes in HD.
• HD lowers mito protein synthesis and higher ribosome occupancy in mito-encoded OXPHOS mRNA.

## Graphical Abstract

**Figure:**
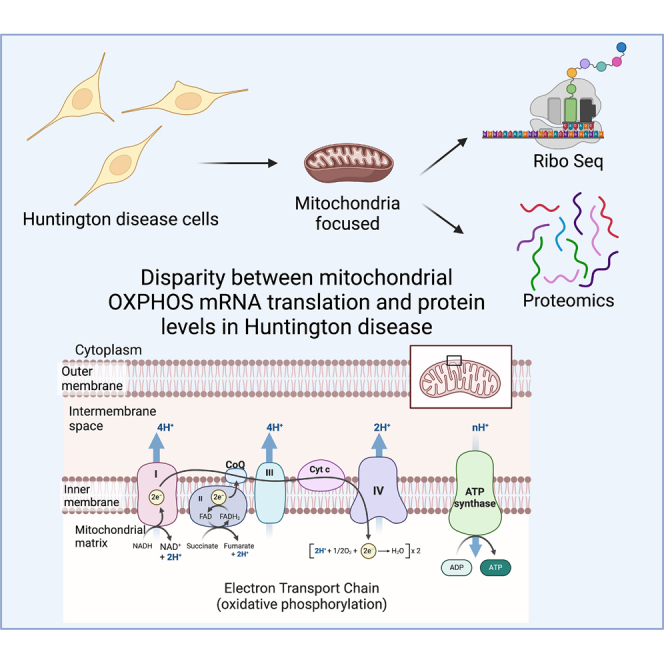

## In Brief

Our data reveal a pervasive dichotomous impact on the ribosome occupancy level and the protein turnover rate in genes associated with mitochondria in HD.

Expansion of the CAG repeat in the huntingtin (HTT) gene causes the motor disturbance, cognitive loss, and psychiatric manifestations of Huntington disease (HD), but the exact mechanism by which mutant HTT (mHTT) induces its pathological effect in the brain remains unclear. Previous studies have reported extensive mitochondrial abnormalities, including decreased complex activities (1, 2, 3, 4, 5, 6, 7, 8), oxidative damage (9, 10), mitochondrial depolarization (11), calcium dyshomeostasis (12, 13), altered biogenesis (14, 15, 16, 17), and mitophagy (18, 19, 20), in HD models and in patient samples. However, despite several investigations, the precise cellular mode(s) of action of mHTT on mitochondria remains controversial. For example, mHTT had no effect on oxidative metabolism and mitochondrial calcium handling in HD (21, 22), whereas mHTT directly interacted with mitochondria and altered mitochondrial proteostasis (23, 24, 25, 26, 27). Consequently, the details of the mechanisms that relate mHTT to mitochondrial defects remain unclear.

Mitochondria are essential organelles that play a vital role in numerous cellular processes, and they contain their own semiautonomous system of gene expression and mRNA translation machineries. The mitochondrial genome codes for components of the ribosomal RNA genes and 22 transfer RNA genes, as well as 13 proteins (28, 29) that participate in the oxidative phosphorylation (OXPHOS) reactions of complexes I, III, and IV. All additional genes required for mtDNA maintenance, replication, transcription, translation, posttranslational modification, transport, assembly, and expression of OXPHOS complexes (II and V) are exclusively encoded by the nucleus (30). Thus, studies of mitochondrial functions, and particularly the translation and assembly of the OXPHOS complexes, are mechanistically challenging, as these complexes are encoded by different genomes. For these reasons, the molecular and biochemical control of mitochondrial complex mRNA translation as well as their possible abnormalities in neurodegenerative diseases remain poorly understood.

The aim of the present study was to subject healthy and HD striatal cells to biochemical, high-resolution ribosome profiling (Ribo-Seq), and tandem mass tag–based mass spectrometry (TMT-MS) investigations to demonstrate mitochondrial translational deficits in HD. Here, we report our analysis of the relative expression levels of mitochondrially encoded mRNA transcripts, ribosome density, and ribosome occupancy in the OXPHOS genes, as well as global proteomics analysis of HD mitochondria.

## Experimental Procedures

### Experimental Design and Statistical Rationale

Ribo-seq/RNA-seq experiments are already published and the n = 3/group was enough to detect statistically significant differences. In tandem mass tag (TMT) labeling–based proteomics study, a total of 15 samples where each genotype was represented by five replicates that were independent rounds of cell cultures prepared separately, from each of the cell line representing the control, HD-het, and HD-homo genotypes. We estimated the variance within each group would be relatively lower since cell line replicates are relatively biologically consistent due to the shared genetic background. Experiments were performed in biological triplicates except for proteomics study. Proteomics studies require higher statistical power due to multiple testing and relatively high variation; therefore, the replications were increased to five samples per group. All statistical tests are explained in the relevant sections.

### Cell Culture

Mouse striatal cells (ST*Hdh*) expressing knock-in WT Htt^exon1^ with seven Gln repeats (control; ST*Hdh*^*Q7/Q7*^) or expressing knock-in mutant human Htt^exon1^ with 111 Gln repeats (HD-het; ST*Hdh*^*Q7/Q111*^, and HD-homo; ST*Hdh*^*Q111/Q111*^) (31) were purchased from Coriell Institute for Medical Research and cultured in 10% fetal bovine serum, Dulbecco's modified Eagle's medium, high glucose, 5% CO_2_, at 33 °C, as described before (32).

#### Immunocytochemistry and Image Analysis

The mouse striatal cells (control, HD-Het, and HD-Homo) were seeded at 60 to 70 percent confluency on glass bottom cell culture dishes. After 24 h, the cells were incubated with vehicle control (dimethyl sulfoxide) and puromycin (100 μg/ml) for 20 min and then washed with cold PBS, followed by fixation using 4% paraformaldehyde for 20 min. After fixation, cells were treated with 0.1 M glycine and permeabilized with 0.1% (v/v) Triton X-100 for 5 min. Subsequently, the cells were blocked with a buffer containing 1% normal donkey serum, 1% bovine serum albumin, and 0.1% Tween 20 in PBS for 1 h at room temperature.

After blocking, the cells were stained overnight at 4 °C using puromycin antibodies (MABE343; 1:10,000). Following this, the cells were incubated for 1 h at room temperature with secondary antibodies labeled with Alexa Fluor 488 (1:1000), along with the nuclear stain 4′,6-diamidino-2-phenylindole. Finally, the stained cells were covered with Fluoromount-G mounting medium. Images were taken using Olympus FV-3000 confocal microscope at 60×. Cytosolic fluorescence integrated density of the confocal micrographs was analyzed using Fiji software (https://imagej.net/software/fiji/). In order to get the integrated fluorescence density of the cytosol, background corrected integrated fluorescence density of the nucleus was subtracted from the background corrected integrated fluorescence density of the whole cell.

### Isolation of Ribosomes (Mitoribosomes and Cytoribosomes) for Profiling

Global RNase foot-printings were performed during three independent rounds of cell cultures (n = 3), as described before (35). For each round of global foot printing, mouse immortalized striatal cells (*i.e.*, control, HD-het, and HD-homo cells) were plated in 15 cm dishes at a confluency of 70%. The following day, the mediums were changed, and after 2 h the cells were incubated with cycloheximide (CHX, 100 μg/ml) for 10 min as in previous studies (33, 34, 35). Cells were then scraped and washed with cold PBS (containing 100 μg/ml CHX) twice. During the second wash, 5% of cells were transferred to new tubes and were lyzed by adding 700 μl of QIAzol lysis reagent. Total RNAs of these samples were isolated using miRNeasy Mini Kit (Qiagen) for total mRNA sequencing. After the second wash, the rest of the cells were lyzed in a lysis buffer containing 20 mM Hepes pH 7.3, 150 mM KCl, 10 mM MgCl_2_, 2 mM DTT, 100 μg/ml CHX, 0.5% v/v Triton X-100, 20 U/ml RNasin, and EDTA free protease inhibitor cocktail (Roche). The cell lysates were passed 20 times through a 26G needle and incubated on ice for 15 min, then centrifuged at 21,000 rpm for 15 min. Supernatants were transferred to new tubes. Equal total RNA amount of each sample was used for global RNase foot printing as follow; for each A260 absorbance unit of the lysates 60 units of RNaseT1 (Thermo Fisher Scientific) and 0.6 μl of RNaseA (Ambion) were added and the samples were incubated at 25 °C for 30 min. RNase-treated samples were immediately loaded on 10 to 50% sucrose gradients and centrifuged at 40,000 RPM (SW41Ti rotor) at 4 °C for 2 h. Gradients were fractionated using a gradient fractionator and UA-6 detector, 254 nm filter (ISCO/Brandel). Fractions containing both 55S (mitoribosomes) and 80S (cytoribosomes) peaks of each sample were collected, and their RNAs were isolated using a miRNeasy Mini Kit (Qiagen).

### Generation of cDNA Libraries from Ribosome Protected mRNAs

The following procedure was performed for all the RNA samples simultaneously, as described before (35). Twenty micrograms of each sample was run on a 15% Tris/Borate/EDTA-Urea gel (Novex) along with 26 and 32 nt RNA markers. The gel containing each sample was excised between two markers. RNAs were extracted from gel pieces by incubating gel slurries with nuclease-free water overnight at 4 °C and precipitated using RNase-free isopropanol and then eluted in nuclease-free water. T4 polynucleotide kinase (NEB) was used to catalyze the addition of 5′ monophosphate and removal of the 3’ phosphate in the RNA fragments to leave a 3′ hydroxyl terminal needed for adapter ligation. RNA was purified using the Zymo clean and conc-5 kit (Zymo Research, Cat. # R1013). Ribosomal RNA was depleted from the samples using TruSeq total RNA rRNA-depletion protocol (Illumina, Cat. #RS-122-2201), and then RNA samples were purified using Agencourt RNAClean XP beads (Beckman-Coulter).

### Generation of cDNA Libraries and Sequencing

NEXTflex small RNA-seq Kit v3 (PerkinElmer) was used to ligate 5′ and 3′ adapters to purified ribosome-protected fragments (RPFs), which then were reverse transcribed and amplified (14 cycles) to generate complementary DNA (cDNA) libraries. Libraries were cleaned up using NEXTflex Cleanup beads, pooled, and sequenced in the NextSeq 500 (V2) using single end 50 bp chemistry at the Scripps Genomic Core.

### Generation of mRNA-Seq Libraries

NEBNext Ultra II Directional kit (NEB, Cat. # E776) with the NEBNext poly(A) mRNA Magnetic isolation module (NEB, Cat. # E7490) was used to generate mRNA-seq libraries. Briefly, 400 ng of high-quality total RNA was used to purify poly(A) mRNA, fragmented, reverse-transcribed with random primers, adapter ligated, and amplified according to manufacturer recommendations. The final libraries were validated on the bioanalyzer, pooled, and sequenced on the NextSeq 500 using paired end 40 bp chemistry.

### Ribo-Seq, RNA-Seq Quality Control, and Mapping the Reads to UCSC Browser

RNAseq reads were trimmed using Cutadapt (36) with the following parameters: -a AGATCGGAAGAGCACACGTCTGAACTCCAGTCA -A AGATCGGAAGAGCGTCGTGTAGGGAAAGAGTGT—minimum-length = 15 –pair-filter = any. For Riboseq reads, 3′ adapters were trimmed using Cutadapt with the following parameters: -a TGGAATTCTCGGGTGCCAAGG—minimum-length 23. The reads were further trimmed using Cutadapt to remove four bases from either side of each read accordingly to the NEXTflex Small RNA trimming instructions (cutadapt -u 4 -u -4). Fastq files were checked for quality control with FastQC. Both RNAseq and Riboseq reads were next mapped to a library of mouse rRNA and tRNA sequences using Bowtie v1.1.2. Any reads mapping to these abundant contaminants were filtered out. Remaining reads were then aligned to the mouse transcriptome with RSEM v1.3.0 (https://deweylab.github.io/RSEM/) (37) using the GRCm38.p5 genome annotation and the comprehensive gene annotation from Gencode (M16 release) as transcriptome reference. Reads with a mapping quality <5 were discarded. Cleaned bam files were converted to bigWig files with Bedtools (38) for visualization using the University of California Santa Cruz (UCSC) Genome Browser. For the euclidian distance analyses, gene expression was quantified with RSEM and comparison plots were generated in R using DESeq2 (https://bioconductor.org/packages/release/bioc/html/DESeq2.html) (39) and ggplot2 packages.

### TMT Quantitative Proteomics and Mass Spectrometry

Protein samples in ∼300 μl of radio-immunoprecipitation assay buffer, were precipitated overnight at −20 °C using 4× ice old acetone (v/v), washed 4 times with ice-cold acetone and then allowed to air dry (Table 1). The protein pellets were subsequently resolubilized in 23 μl of 5% SDS (v/v) and processed for digestion using micro-S-Traps (ProtiFi) according to the manufacturer’s instructions. Briefly, proteins in 5% SDS were reduced with 1.43 μl of 120 mM Tris (2-carboxyethyl) phosphine hydrochloride at 56 °C for 20 min, followed by alkylation using 1.43 μl of 500 mM methyl methanethiosulfonate for 20 min at ambient temperature. Finally, 4 μg of sequencing-grade trypsin (Promega) was added and the mixture was incubated for 1 h at 47 °C. Following this incubation, 40 μl of 50 mM triethylammonium bicarbonate was added to the S-Trap, and the peptides were eluted using centrifugation. Elution was repeated once. A third elution using 35 μl of 50% acetonitrile was also performed, and the eluted peptides dried under a vacuum. The peptides were then resolubilized in 50 μl of 50 mM triethylammonium bicarbonate pH 8.5 and assayed using the Pierce Quantitative Fluorometric Peptide Assay (ThermoFisher Scientific). Four micrograms of peptides/samples were labeled with TMT labels (16-plex) according to the manufacturer’s instructions (ThermoFisher Scientific), and pooled. The multiplexing strategy is provided below. The pooled, plexed samples were then dried under vacuum, resolubilized in 1% trifluroacetic acid, and finally desalted using 2 μg capacity ZipTips (Millipore) according to the manufacturer instructions. Peptides were then on-line eluted into a Fusion Tribrid mass spectrometer (ThermoFisher Scientific) from an EASY PepMap RSLC C18 column (2 μm, 100 Å, 75 μm × 50 cm, Thermo Fisher Scientific), using a gradient of 5 to 25% solvent B (80/20 acetonitrile/water, 0.1% formic acid) in 180 min, followed by 25 to 44% solvent B in 60 min, 44 to 80% solvent B in 0.1 min, a 5 min hold of 80% solvent B, a return to 5% solvent B in 0.1 min, and finally a 10 min hold of solvent B. All flow rates were 250 nl/min delivered using an nEasy-LC1000 nano liquid chromatography system (ThermoFisher Scientific). Solvent A consisted of water and 0.1% formic acid. Ions were created at 2.4 kV using an EASY Spray source (ThermoFisher Scientific) held at 50 °C. A synchronous precursor selection-MS3 mass spectrometry method was selected based on the work of Ting *et al.* (40). Scans were conducted between 380 to 2000 m/z at a resolution of 120,000 for MS1 in the Orbitrap mass analyzer at an AGC target of 4E5 and a maximum injection of 50 msec. Collision induced dissociation was then performed in the linear ion trap of peptide monoisotopic ions with charge 2 to 8 above an intensity threshold of 5E3, using a quadrupole isolation of 0.7 m/z and a collision induced dissociation energy of 35%. The ion trap AGC target was set to 1.0E4 with a maximum injection time of 50 ms. Dynamic exclusion duration was set at 60 s and ions were excluded after one time within the ± 10 ppm mass tolerance window. The top 10 MS2 ions in the ion trap between 400 to 1200 m/z were then chosen for higher-energy C-trap dissociation at 65% energy. Detection occurred in the Orbitrap at a resolution of 60,000 and an AGC target of 1E5 and an injection time of 120 ms (MS3). All scan events occurred within a 3-s specified cycle time. The analysis was performed at The Herbert Wertheim UF Scripps Institute for Biomedical Innovation & Technology, Mass Spectrometry and Proteomics Core Facility, SCR_023576.

**Table 1:** TMT labeling table

Plex	TMTpro-126	TMTpro-127N	TMTpro-127C	TMTpro-128N	TMTpro-128C	TMTpro-129N	TMTpro-129C	TMTpro-130N	TMTpro-130C	TMTpro-131N	TMTpro-131C	TMTpro-132N	TMTpro-132C	TMTpro-133N	TMTpro-133C	TMTpro-134N
	Homo 1	Het 1	Homo 2	Het 2	Homo 3	Het 3	Homo 4	Het 4	Homo 5	Het 5	NOT USED	Cont 1	Cont 2	Cont 3	Cont 4	Cont 5

### Proteomic Data Processing and Statistical Analysis

Quantitative analysis of the TMT experiments was performed simultaneously with protein identification using Proteome Discoverer 2.5 software (https://www.thermofisher.com/us/en/home/industrial/mass-spectrometry/liquid-chromatography-mass-spectrometry-lc-ms/lc-ms-software/multi-omics-data-analysis/proteome-discoverer-software.html). The precursor and fragment ion mass tolerances were set to 10 ppm, 0.6 Da, respectively), enzyme was trypsin with a maximum of 2 missed cleavages and UniprotKB Mouse Proteome UP000000589 Strain C57BL_6J FASTA file and common contaminant FASTA file used in Sequest searches (http://fields.scripps.edu/sequest/) (55,749 proteins in total). The impurity correction factors obtained from Thermo Fisher Scientific for each kit was included in the search and quantification. The following settings were used to search the data; dynamic modifications; Oxidation/+15.995 Da (M), Deamidated/+0.984 Da (N, Q), and static modifications of TMTpro/+304.207 Da (K) (N Terminus, K), MMTS/+45.988 Da (C). Scaffold Q+ (version Scaffold_5.0.0, Proteome Software Inc, Portland, (https://www.proteomesoftware.com/products/scaffold-qs) OR) was used to TMTpro peptide and protein identifications. Peptide identifications with 99.0% < probability was accepted to achieve an false discovery rate (FDR) less than 1.0% by the percolator posterior error probability calculation (41). Proteins with 93.0%< probability were accepted to achieve an FDR less than 1.0% and contained at least 1 identified peptide. Protein probabilities were assigned using the Protein Prophet algorithm (42), and default Scaffold settings were employed, with the exception of the Blocking level, which was set to Unique Samples. Mean values were utilized for averaging, and normalization was applied according to Oberg et al. (43). Of 12,543 spectra in the experiment at the given thresholds, 11,175 (89%) were included in quantitation. Differentially expressed proteins were determined by first applying ANOVA to obtain unadjusted *p* values then corrected by Benjamini-Hochberg (FDR < 0.05). The proteins that passed this criterion were used to compare Het *versus* Control and Homo *versus* Control *via* t-test (*p* < 0.05) in Scaffold. The ANOVA and *t* test results were exported from Scaffold and combined using JMP Pro 17.1.0 SAS Institute Inc, Cary, NC, 1989 to 2023 (https://www.jmp.com/en_us/home.html). The analysis was performed at The Herbert Wertheim UF Scripps Institute for Biomedical Innovation & Technology, Bioinformatics and Statistics Core Facility (RRID:SCR_023048). The proteomics data, analysis results, and method were uploaded to MassIVE with the ID number MSV000092843/PXD045329.

### STRING Analysis Parameters

Network nodes indicate proteins whose splice isoforms or posttranslational modifications have collapsed, *i.e.*, each node represents all proteins produced by a single protein-coding gene locus. And the edges reflect protein-protein relationships, which are intended to be particular and meaningful, *i.e*., proteins that collaborate to perform a common function; this does not necessarily imply that they are physically bound to one other. The network type is full-string network, and the network's meaning is evidence-based. Active interaction sources include text mining, experiments, databases, coexpression, neighborhoods, gene fusion, and cooccurrence. The minimum needed interaction score was set to medium confidence (0.400).

### Isolation of Cytoplasm and Mitochondria

Cytoplasm and mitochondria are separated using mitochondria isolation kit (ThermoFisher Scientific, Cat #. 89874). In brief, control, HD-het and HD-homo cells were grown in 10 cm dish to 80 to 90% confluency. The cells were pelleted, and the mitochondria and cytoplasm were separated as described in the manufacturer’s protocol. Around 5 mg of mitochondrial proteins per sample was provided for TMT-MS analysis. Around 20 mg of proteins from cytoplasm and mitochondria fractions from cells treated with puromycin (20 μM) was probed in Western blotting. For *in vitro* protein synthesis experiments the isolated mitochondria were treated with 10 μM of puromycin for 10 min before proceeding to Western blotting.

### Western Blot Analysis

The equal protein amounts were loaded for Western blots analysis using antibodies to detect indicated endogenous proteins, as described before (35). The puromycin antibody (MABE343, 1:10,000) was from Millipore; the GAPDH antibody (sc365062, 1:5000) was from Santa Cruz; and the antibodies against succinate dehydrogenase (ubiquinone) flavoprotein subunit (CST 11998, 1:5000) and mTOR (CST 2983, 1:2000) were from cell signaling technology.

### Statistical Analysis

Data were expressed as mean ± SEM as indicated. Experiments were performed on biological triplicates. Statistical analysis was performed with a Student’s *t* test or one-way ANOVA followed by Dunnett’s or Benjamini-Hochberg multiple comparison test.

## Results

### Mitochondrial Translation is Diminished in HD

**Fig. 1:**
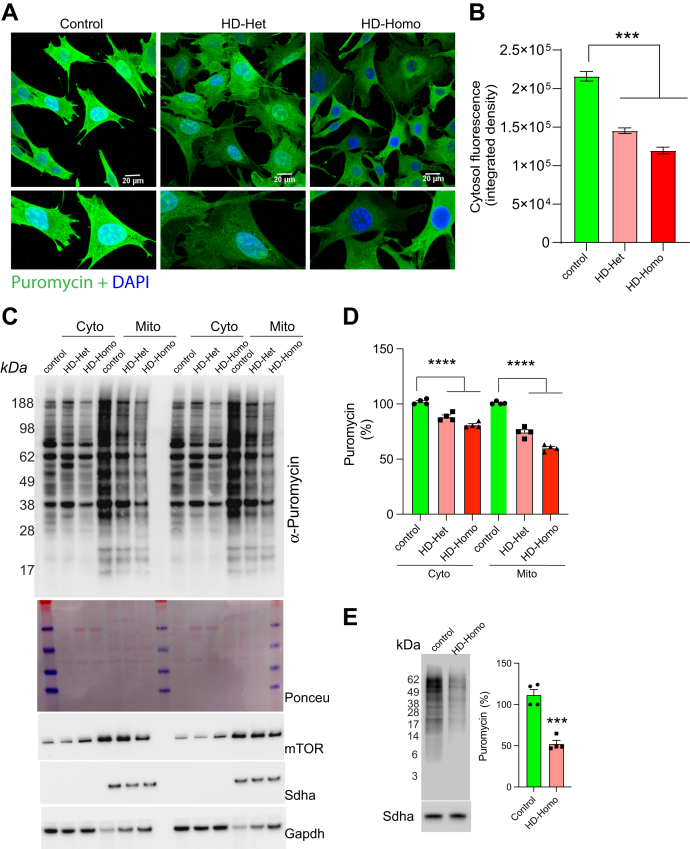
**Mitochondrial protein synthesis is diminished in HD cells.***A*–*D*, representative immunocytochemistry (*A*) and quantification of cytosolic integrated fluorescence density (*B*) of the confocal micrographs from (*A*). Error bar represents ± SEM (n = 3, total control cells 40, HD-Het 50 cells and HD-Homo 50 cells), ∗∗∗*p* < 0.001 by Student’s *t* test. Immunoblots (*C*) of metabolic labeling of protein synthesis, using puromycin, and its quantification in cytoplasm and mitochondrial fractions of control, HD-het and HD-homo cells. *D*, Ponceau staining of the blots and other indicated proteins is shown. Gapdh and Sdha are used as normalization control for cytoplasm and mitochondria respectively. Error bar represents ± SEM (n = 4, independent experiments) ∗∗∗∗*p* < 0.0001 by ANOVA. *E*, representative puromycin immunoblot in isolated mitochondria and its quantification in control and HD-homo cells. Error bar represents ± SEM (n = 4, independent experiments), ∗∗∗*p* < 0.001 by Student’s *t* test. HD, Huntington's disease.

Our prior work revealed that overall protein synthesis is reduced in HD, as measured by the incorporation of puromycin, a t-RNA analog that undergoes a covalent interaction with growing peptides. Incorporated puromycin is detected using antibodies in immunocytochemistry or Western blotting. We used well-established knock-in mouse striatal cell lines that express full-length WT control (polyQ7) HTT and one (HTT-het) or two (HTT-homo) copies of mutant (polyQ111) HTT. The striatal cells were treated with puromycin, and we then analyzed overall protein synthesis by immunocytochemistry using puromycin antibody. Consistent with a prior report (35), we found a substantial reduction in puromycin incorporation in the HD-het and HD-homo cells compared to the control cells (Fig. 1*A*).

We then distinguished protein synthesis between the cytoplasm and mitochondrial compartments by treating the cells with puromycin, biochemically separating the cells into cytoplasmic and mitochondrial fractions, and then measuring puromycin incorporation by Western blotting (Fig. 1*B*). This approach revealed a significant reduction in protein synthesis in both the cytoplasmic and mitochondrial fractions (Fig. 1*C* and *D*). We also determined whether mitochondrial translation turnover was independent of cytoplasmic turnover in HD by isolating mitochondria from control and HD cells and investigating *in vitro* puromycin incorporation by the isolated mitochondria. Translational turnover was strongly diminished in the isolated mitochondria compared to the control cells (Fig. 1*E*). Taken together, these results provided the first indication that mitochondrial translation is diminished in the HD cell model.

### Ribosome Profiling of Ribosome-Protected Fragments from 55S and 80S Ribosomes

**Fig. 2:**
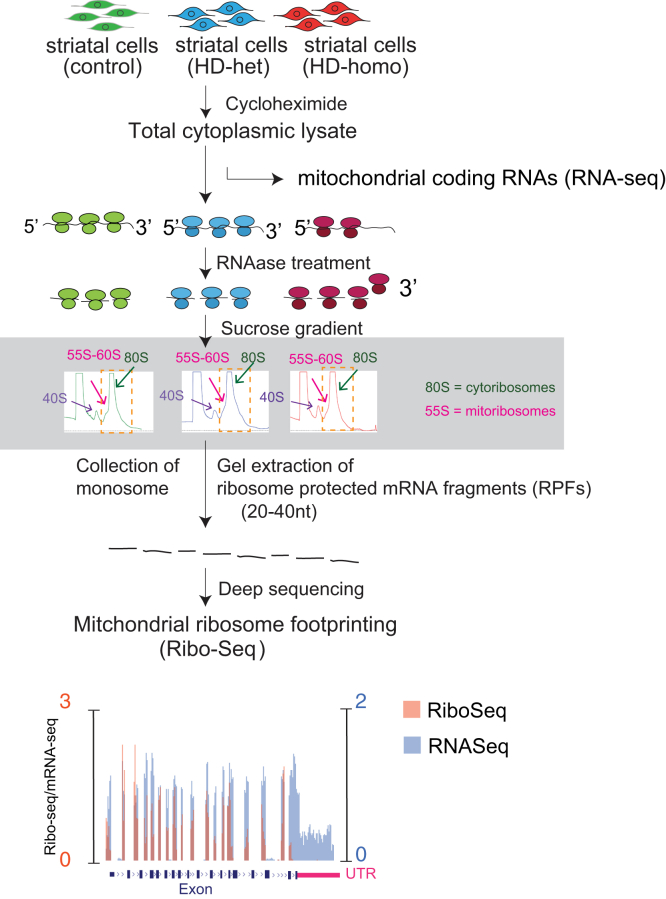
**Ribosome profiling of ribosome-protected fragments from 55S and 80S ribosomes.** RNAase digested polysomes collected as 80S peaks most likely comprised of mitoribosomes with lower sedimentation coefficients (∼55S, *brown arrow*).

We explored whether a correlation exists between the reduced protein synthesis in mitochondria affected by HD and changes in ribosome occupancy by examining the results of our comprehensive analysis of Ribo-Seq and RNA-seq data obtained from control, HD-het, and HD-homo cells. Three replicates of control, HD-het, and HD-homo mutant striatal cells were subjected to ribosome profiling, and the fractions containing 55S ribosomes (mitoribosomes) and 80S ribosomes (cytoribosomes) were collected for Ribo-Seq and matching RNA-Seq (Fig. 2), as described previously (35). The use of multiple quality control measures generated a high-quality global Ribo-Seq library of control, HD-het, and HD-homo cells (35).

We used this Ribo-Seq library for the specific analysis of the ribosome profiles of mitochondrially encoded mRNAs. We combined the profiles from the triplicate experiments, uploaded as a track hub in the UCSC Genome Browser 42, and overlaid ribosome protected fragments (RPFs, orange) and mRNA abundance (mRNA, blue). We then estimated the ribosome occupancy as the ratio between the RPF abundance and mRNA abundance and for each gene (RPF/mRNA) from the raw read counts from the UCSC browser (Fig. 2) (35).

### Ribosome Occupancy on the Mitochondrially Encoded OXPHOS Genes is Increased in HD

**Fig. 3:**
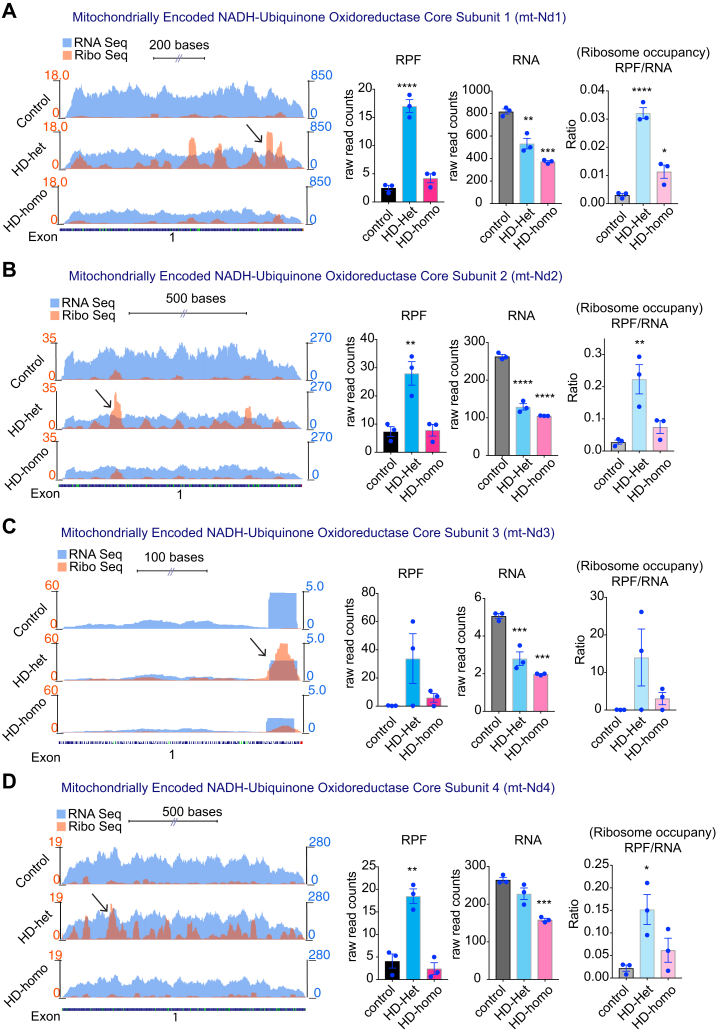
**Ribosome occupancy on the mitochondrially encoded ND1, ND2, ND3 and ND4 genes.***A*–*D*, representative graphs showing overlay of mitochondrially encoded transcripts Ribo-Seq (RPF)/mRNA-Seq and their corresponding average individual raw reads for RPF and mRNA and the ratio of RPF/mRNA (ribosome occupancy) from the triple experiments for mt-ND1 (*A*), mt-ND2 (*B*), mt-ND3 (*C*), and mt-ND4 (*D*), extracted from UCSC Genome Browser. Error bar represents mean ± SEM (n = 3 independent experiments), ∗∗∗∗*p* < 0.0001, ∗∗∗*p* < 0.001, ∗∗*p* < 0.01, ∗*p* < 0.05, one-way ANOVA followed by Dunnett’s multiple comparison test. RPF, ribosome-protected fragment; UCSC, University of California Santa Cruz.

**Fig. 4:**
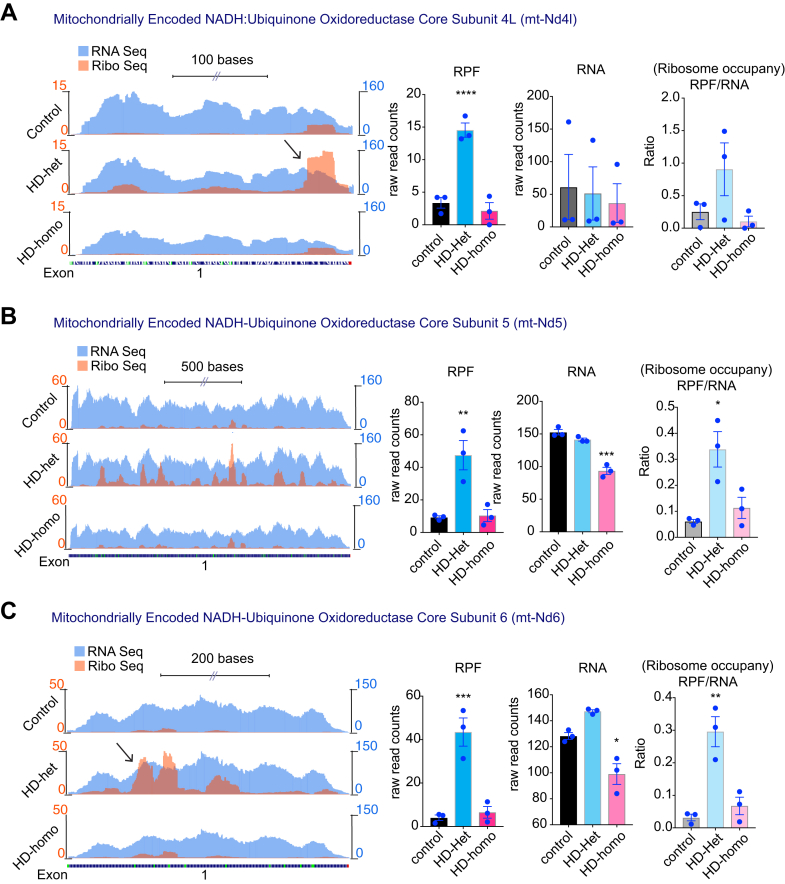
**Ribosome occupancy on the mitochondrially encoded ND4L, ND5 and ND6 genes.***A–C,* Representative graphs showing overlay of mitochondrially encoded mRNA transcripts Ribo-Seq (RPF)/mRNA-Seq and their corresponding average individual raw reads for RPF and RNA and the ratio of RPF/RNA (ribosome occupancy) from the triple experiments for mt-Nd4l (*A*), mt-Nd5 (*B*), and mt-Nd6 (*C*) extracted from UCSC Genome Browser. Error bar represents mean ± SEM (n = 3 independent experiments), ∗∗*p* < 0.01, ∗*p* < 0.05, one-way ANOVA followed by Dunnett’s multiple comparison test. RPF, ribosome-protected fragment; UCSC, University of California Santa Cruz.

**Fig. 5:**
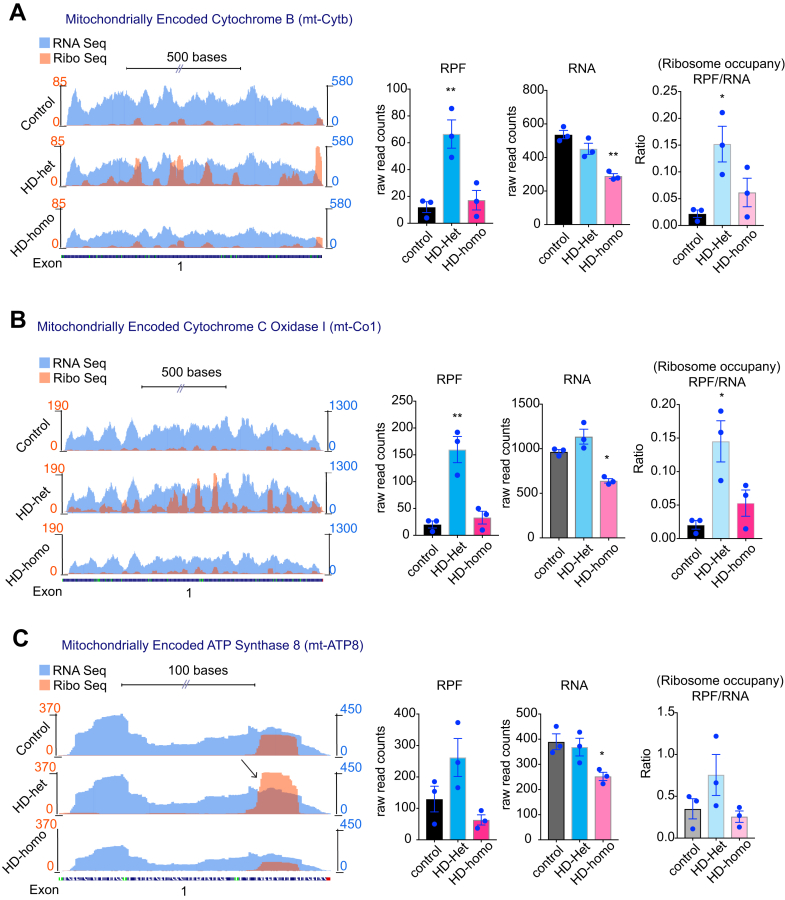
**Ribosome occupancy on the mitochondrially encoded CyB, Co1 and ATP8 genes.***A–C,* Representative graphs showing overlay of mitochondrially encoded mRNA transcripts Ribo-Seq (RPF)/mRNA-Seq and their corresponding average individual raw reads for RPF and RNA and the ratio of RPF/RNA (Ribosome occupancy) from the triple experiments for mt-Co1 (*A*), mt-CyB (*B*), and mt-ATP8 (*C*) extracted from UCSC Genome Browser. Error bar represents mean ± SEM (n = 3 independent experiments), ∗∗*p* < 0.01, ∗*p* < 0.05, one-way ANOVA followed by Dunnett’s multiple comparison test. RPF, ribosome protected fragment; UCSC, University of California Santa Cruz.

Among 13 mitochondrially encoded OXPHOS mRNA transcripts (mt-OXPHOS transcripts), we observed ribosome occupancy for 11 mt-OXPHOS mRNA transcripts. The complex I subunits mt-Nd1, mt-Nd2, mt-Nd3, mt-Nd4, mt-Nd4l, mt-Nd5, and mt-Nd6 showed high RPF, low mRNA, and high ribosome occupancy (RPF/mRNA) in HD cells compared to the control cells. The ribosome occupancy on these transcripts was also much higher in HD-het than in HD-homo cells [Figs. 3, *A*–*D*, and 4, *A*–*C*]. Mt-CyB, the only mitochondrially encoded complex III subunit, also showed high RPF and high ribosome occupancy (Fig. 5*A*). Differential ribosome density was also evident throughout the transcripts, with mt-Nd1, mt-Nd3, mt-Nd4l, and mt-Atp8 transcripts exhibiting a preferential accumulation of ribosomes toward the 3′ region, whereas mt-Nd2, mt-Nd4, and mt-Nd6, exhibited ribosome accumulation toward the 5' region (Fig. 3, Fig. 4, Fig. 5, arrow).

**Supplemental Figure S1:**
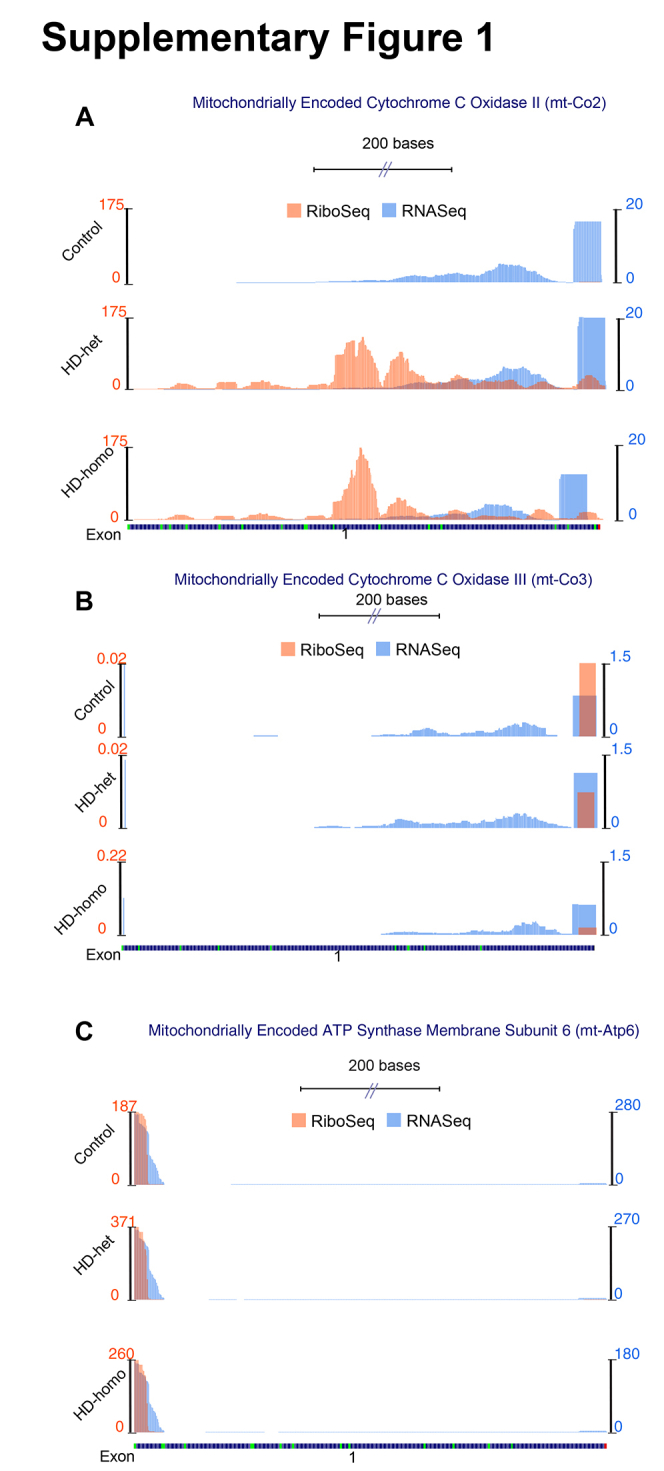
Representative graphs showing overlay of mitochondrially-encoded transcripts Ribo-Seq (RPF)/mRNA-Seq and their corresponding average individual raw reads for RPF and mRNA and the ratio of RPF/mRNA (Ribosome occupancy) from the triple experiments for mt-Co2 (*A*), mt-Co3 (*B*), and mt-Atp6 (*C*) extracted from UCSC Genome Browser.

The complex IV subunit mt-Co1 and the complex V subunit mt-Atp8 showed high RPF and a significant trend toward higher ribosome occupancy in the HD-het and HD-homo cells (Fig. 5, *B* and *C*). The ribosome profiling data for the three remaining mt-OXPHOS transcripts (mt-Co3, mt-Co2, and mt-Atp6) indicated a consistent pattern of increased ribosome occupancy in cells affected by HD (supplemental Fig. S1). However, we were unable to obtain statistically significant differences for these transcripts as either RPF or mRNA reads were discernible in one or more replicates of the Ribo-Seq experiments (https://genome.ucsc.edu/cgi-bin/hgTracks?hubUrl=Https://de.cyverse.org/anon-files/iplant/home/rmi2lab/Hub_Collaborations/Srini/hub.txt&genome=mm10).

In summary, Ribo-Seq analysis suggested that most of the mt-OXPHOS mRNA transcripts exhibited an increased level of ribosome occupancy, along with changes in ribosome density, in cells affected by HD. These observations imply a significant disruption in the mobility of ribosomes on mitochondrially encoded transcripts in HD.

### Ribosome Occupancy on the Nuclear-Encoded OXPHOS Genes Remains Largely Unaltered in HD

**Fig. 6:**
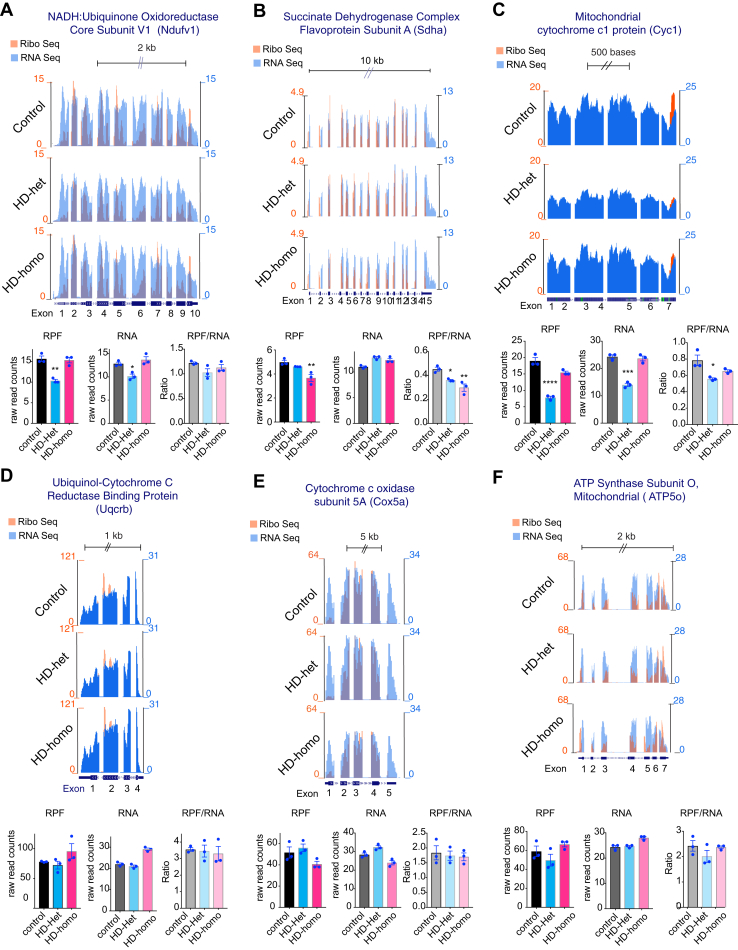
**Ribosome occupancy on the nuclear-encoded OXPHOS genes remains largely unaltered in HD.***A–F,* Representative graphs showing overlay of mitochondrially encoded mRNA transcripts ribosome occupancy (Ribo Seq/RNA Seq or RPF/RNA) and their corresponding average individual raw reads for RPF and RNA and the ratio of RPF/RNA from the triplicate experiments for Ndufv1 (*A*), Sdha (*B*), Cyc1 (*C*), Uqcrb (*D*), Cox5a (*E*), Atp5o (*F*) extracted from UCSC Genome Browser. Error bar represents mean ± SEM (n = 3 independent experiments), ∗∗∗∗*p* < 0.0001, ∗∗∗*p* < 0.001, ∗∗*p* < 0.01, ∗*p* < 0.05, one-way ANOVA followed by Dunnett’s multiple comparison test. RPF, ribosome protected fragment; UCSC, University of California Santa Cruz.

The higher ribosome occupancy in subunits encoded by mitochondria could change the ribosome occupancy in subunits encoded by the nucleus that makes up the respiratory chain. This correlation is likely important in upholding the equilibrium between the influx of nuclear-encoded respiratory components and the continuous presence of mitochondria-encoded chain subunits. Our examination of the Ribo-Seq of selected nuclear-encoded mitochondrial complexes in control and HD cells revealed that the subunits of complex I, namely Ndufv1, exhibited a distribution of ribosomes throughout all 10 exons. Nevertheless, in the HD-het cells, the RPF declined significantly as a result of lower RNA levels. Conversely, in HD-homo cells, the RPF remained unchanged compared to the control group. (Fig. 6*A*). No discernible variations were detected in the total ribosome occupancy (RPF/RNA) of the Ndufv1 mRNA between the control and HD cells (Fig. 6*A*).

A substantially lower ribosome occupancy was also observed for the complex II subunit Sdha in HD cells than in control cells (Fig. 6*B*). By contrast, the ribosome occupancy levels for the remaining subunits of complex II (Sdhb, Sdhc, and Sdhd) did not differ significantly between the control and HD cells (data not shown). The ribosome occupancy of complex III subunit Cyc1 was diminished specifically in the HD-het cells compared to the controls (Fig. 6*C*), whereas the nuclear-encoded complex III subunit Uqcrb or complex IV subunits (Cox5a) showed no differences in ribosome occupancy between the control and HD cells (Fig. 6, *D* and *E*). The ribosome occupancy on the subunits of complex V (ATP5o) was unchanged (Fig. 6*F*). Analysis of the remaining 55 transcripts that encode nuclear-encoded OXPHOS transcripts revealed no substantial ribosome occupancy, in contrast to the transcripts that encode mitochondria-encoded OXPHOS proteins (data can be found in https://genome.ucsc.edu/cgi-bin/hgTracks?hubUrl=Https://de.cyverse.org/anon-files/iplant/home/rmi2lab/Hub_Collaborations/Srini/hub.txt&genome=mm10).

This finding indicates high ribosome occupancy levels for mitochondrial OXPHOS mRNA transcripts in cells affected by HD, whereas the ribosome occupancy of nuclear-encoded OXPHOS transcripts is largely unaltered.

### Quantitative Mitochondrial Proteome Analysis of Control and HD Striatal cells

**Fig. 7:**
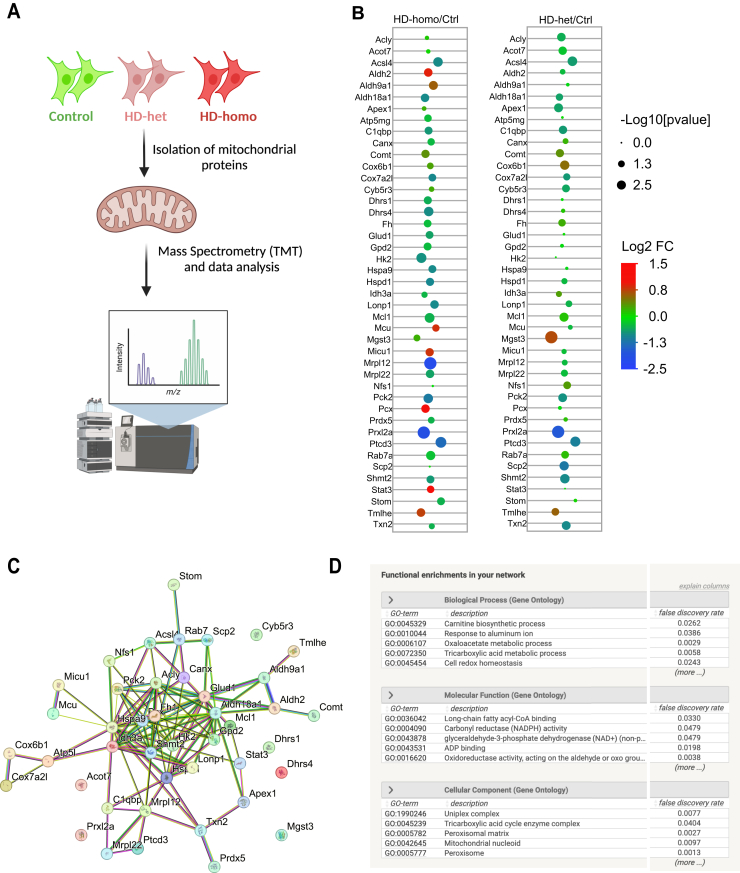
**TMT-MS/MS proteomics of isolated mitochondria from control and HD cells.***A*–*D*, TMT-MS/MS proteomics of isolated mitochondria from control and HD cells. *A*, a schematic flow chart of the TMT-MS/MS proteomics experiment. *B*, *dot plots* of 43 mitochondrial proteins, that were differentially regulated in HD-het or HD-homo genotypes when compared to ctrl (control), were depicted where the size of the dot was proportional to −log10 transformed *p* values and color of the *dot* indicated log2 fold change. *C* and *D*, the STRING network view of mitochondrial proteins identified by TMT-MS/MS analysis shows a comprehensive overview of the anticipated relationships within a specific set of proteins (*C*) and an enrichment of the biological process (*D*). TMT-MS/MS data acquired from n = 5 independent experiments. TMT-MS data were analyzed with ANOVA, with multiple tests considered by controlling FDR with the Benjamini-Hochberg method (FDR < 0.05) in Scaffold. FDR, false discovery rate; HD, Huntington disease; TMT-MS/MS, tandem mass tag–based mass spectrometry.

Our Ribo-Seq analysis provided novel insight into the translation status of mitochondrial OXPHOS transcripts; however, it did not reveal the true *in vivo* status of mitochondrial proteins. We addressed this limitation by quantifying mitochondrial proteins using TMT-based MS analysis. Mitochondria were isolated from control, HD-het, and HD-homo striatal cells (n = 5 independent experiments) and subjected to multiplex TMT relative quantitative proteomics analyses (Fig. 7*A*). A total of 2471 protein groups were identified based on FDR of 1% (Data Sheet 1), with significant differences observed in 490 of them (Data Sheet 2), as determined by ANOVA analysis (Benjamini-Hochberg, FDR < 0.05). As approximately 1000 to 1500 mitochondria-associated proteins are encoded in the nucleus (44, 45, 46), this result is not surprising. Within the TMT data, we found significant differences in approximately 43 mitochondrial proteins (23 at *p* < 0.05 for HD-het *versus* control comparison and 32 at *p* < 0.05 for HD-homo *versus* control comparison) (Data Sheet 3), using mitocarta3 software (http://www.broadinstitute.org/mitocarta) (47). The fold changes and the *p* values were used to contrast the 43 mitochondrial proteins across het/control and homo/control comparison (Fig. 7*B*). Mrpl12, a mitochondrial ribosomal protein, was significantly lower only in HD-homo. However, peroxiredoxin like 2A, an antioxidant; Ptcd3, a mitochondrial RNA-binding protein; and Pck2, an important enzyme in gluconeogenesis, were lower in both HD-het and HD-homo. In the same way, Nfs1 (an enzyme that changes L-cysteine to L-alanine) and Scp2 (which moves cytoplasmic lipid hydroperoxides to mitochondria) are only upregulated in HD-het. On the other hand, Comt (which breaks down catecholamines), Tmlhe (which makes carnitine), and Mgst3 (which oxidizes hydroxy-fatty acids) are upregulated in both HD-het and HD-homo. Thus, TMT-based proteomics for the first time identifies mitochondrial proteins that are changed in a mHTT allele-specific way (Fig. 7*B*, Data Sheet 3).

The highest ranking mitochondria-associated proteins, based on STRING pathway analysis, exhibited a distinct correlation that was both unique and significant, indicating that these proteins collectively contributed to a similar biological function (Fig. 7*C* and *D*). They revealed the top biological processes (*e.g.*, carnitine biosynthetic process, TCA cycle, and cell redox homeostasis), molecular functions (*e.g.*, long-chain fatty acyl-CoA binding, NADPH activity, and ADP binding), and cellular components (Uniplex complex, TCA complex, and peroxisome) (Data Sheet 3) that are dysregulated in HD.

Overall, using TMT-MS, we discovered more widespread changes in mitochondrial proteomics in HD cells.

### Disparity Between the Continuous Translation of Mitochondrial mRNA and the Protein Levels in HD

**Fig. 8:**
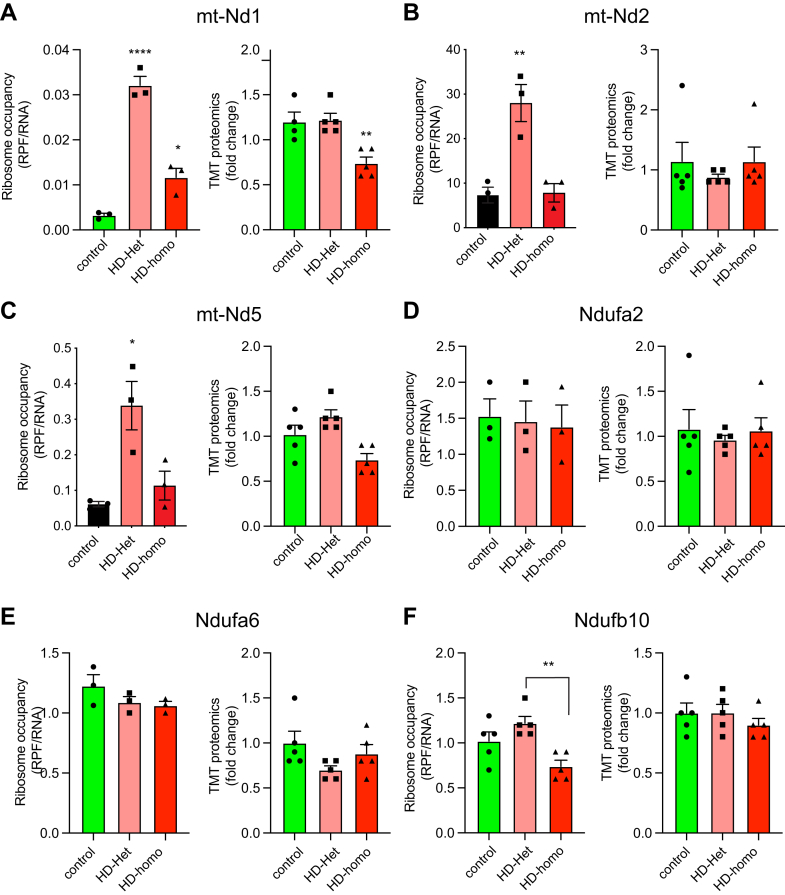
**Comparison of ribosome occupancy (RPF/mRNA) and TMT-MS/MS of selected targets from control and HD cells.***A*–*F*, comparison of ribosome occupancy (RPF/mRNA) and TMT-MS/MS of selected targets from control and HD cells. Representative graphs showing overlay of indicated transcripts for ribosome occupancy (RPF/mRNA) (n = 3 experiments) extracted from UCSC Genome Browser and corresponding TMT-MS ratio of fold change (n = 5 experiments) derived from Scaffold for mt-nd1 (*A*), mt-nd2 (*B*), mt-nd5 (*C*), Ndufa2 (*D*), Ndufa6 (*E*), Ndufa10 (*F*). Error bar represents mean ± SEM ∗∗∗∗*p* < 0.0001, ∗∗*p* < 0.01, ∗*p* < 0.05, one-way ANOVA followed by Dunnett’s multiple comparison test. RPF, ribosome protected fragment; TMT-MS, tandem mass tag–based mass spectrometry; UCSC, University of California Santa Cruz.

We examined whether the Ribo-Seq data correlated with the proteomics data by comparing the differences between ribosome occupancy (RPF/RNA) and protein presence (TMT-MS/MS) for mitochondrial OXPHOS proteins. The mitochondria-encoded OXPHOS protein mt-nd1 showed high ribosome occupancy in HD-het and HD-homo cells; however, the protein level was unaltered in HD-het cells but was significantly diminished in the HD-homo cells compared to the control cells (Fig. 8*A*). Mt-Nd2 and mt-Nd5 exhibited a substantial increase in ribosome occupancy in the HD-het cells but showed no notable variations in their protein levels between the three groups (Fig. 8, *B* and *C*). One point to note is that TMT MS/MS analysis was unable to identify all the mitochondrial OXPHOS proteins (*e.g.*, mt-Nd3, mt-Nd4, mt-Nd4l, mt-Co1, and mtATP8). This limitation could reflect the low abundance of these proteins and the inherent bias of data-dependent acquisition analysis toward more abundant proteins.

**Fig. 9:**
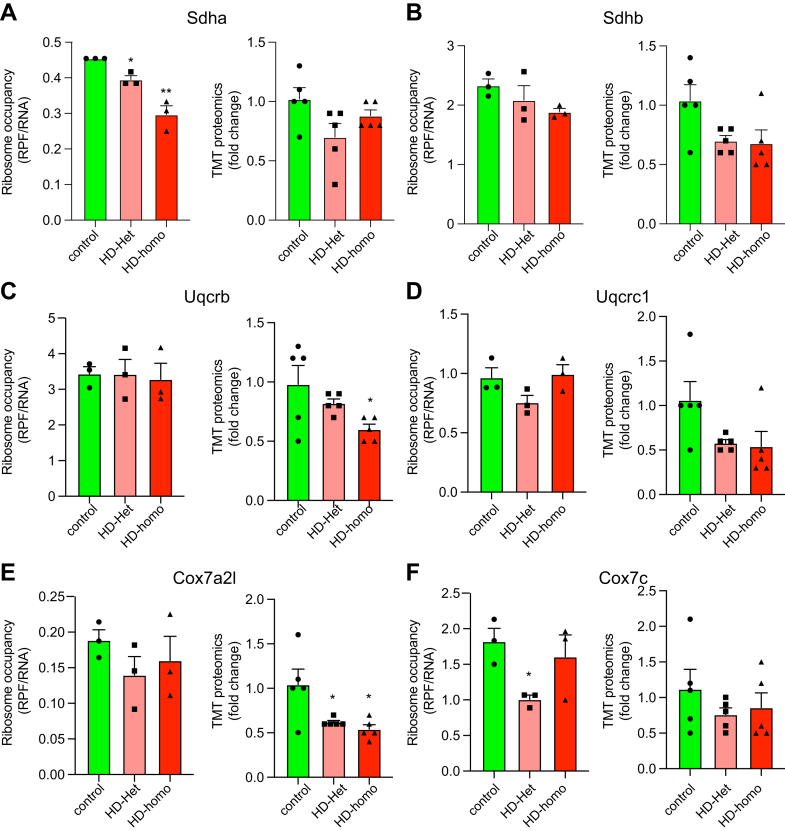
**Reduction in ribosome occupancy in HD cells with no change in protein levels.***A*–*F*, comparison of ribosome occupancy (RPF/mRNA) and TMT-MS/MS of selected targets from control and HD cells. Representative graphs showing overlay of indicated transcripts for ribosome occupancy (RPF/mRNA) (n = 3 experiments) extracted from UCSC Genome Browser and corresponding TMT-MS ratio of fold change (n = 5 experiments) derived from Scaffold for Sdha (*A*), Sdhb (*B*), Uqcrb (*C*), Uqcrc1 (*D*), Cox7a2l (*E*) and Cox7c (*F*). Error bar represents mean ± SEM, ∗∗*p* < 0.01, ∗*p* < 0.05, one-way ANOVA followed by Dunnett’s multiple comparison test. HD, Huntington disease; RPF, ribosome protected fragment; TMT-MS, tandem mass tag–based mass spectrometry; UCSC, University of California Santa Cruz.

We also determined the protein levels of nuclear-encoded OXPHOS proteins. The mitochondrial complex 1 proteins Ndufa2 and Ndufa6 showed no differences in ribosome occupancy or protein levels between the control and HD cells (Fig. 8, *D* and *E*). In the HD-homo cells, the Ndufb10 subunit displayed a slight decrease in ribosome occupancy compared to the HD-het cells, but no change was evident in the protein levels between the 2 cell types (Fig. 8*F*). Within the subset of nuclear-encoded complex II subunits, we observed a notable inconsistency between ribosome occupancy and TMT proteomics in the case of Sdha. Specifically, we observed a large reduction in ribosome occupancy in HD cells, whereas the protein levels were unchanged (Fig. 9*A*). By contrast, no statistically significant alterations were detected for the ribosome occupancy of sdhb. However, a discernible tendency toward a reduction in protein levels was apparent in the HD-het and HD-homo cells (Fig. 9*B*). Interestingly, among the complex III proteins, neither uqcrb nor uqcrc1 showed alterations in ribosome occupancy, whereas their protein levels were significantly reduced (Fig. 9, *C* and *D*). Similarly, nuclear-encoded complex IV, cox7a2l, showed no change in ribosome occupancy, but it demonstrated a noteworthy reduction in protein level (Fig. 9*E*). By contrast, the Cox7c subunit exhibited a significant decrease in ribosome occupancy in HD-het, although the protein levels were unaffected (Fig. 9*F*). These comparative data provide the first suggestion that a disparity exists between the ongoing translation of mRNA and the levels of proteins observed in HD. Examining the processes behind this disparity and its involvement in the development of diseases has the potential to provide new perspectives on the mechanisms and targets for therapeutic interventions.

### Widespread Proteome Alteration in HD Mitochondria

**Fig. 10:**
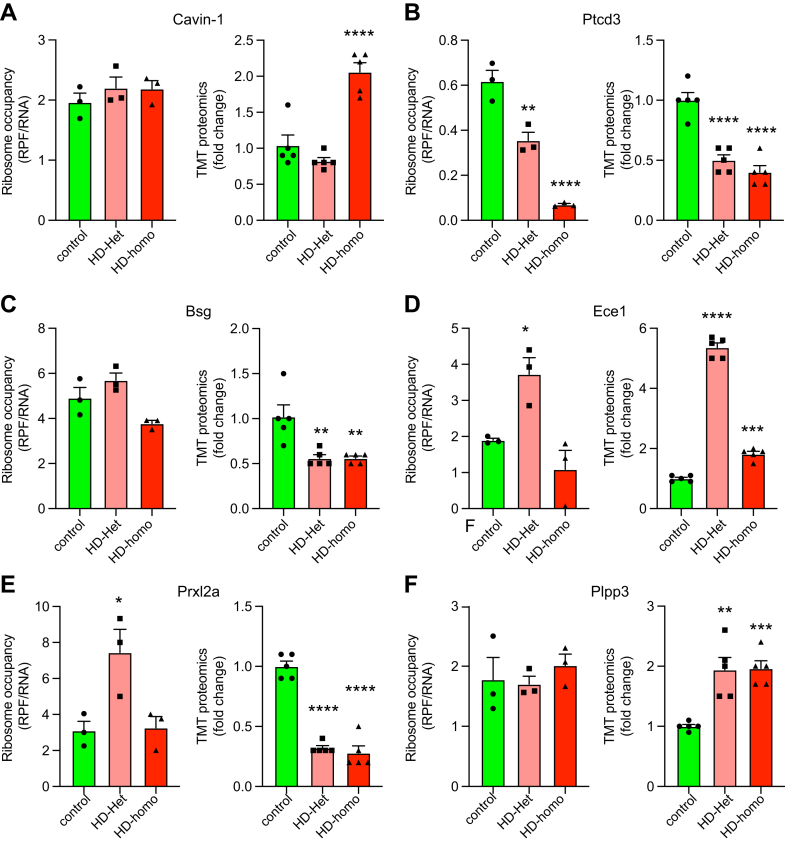
**Widespread proteome alteration in HD mitochondria.***A*–*F*, comparison of ribosome occupancy (RPF/mRNA) and TMT-MS/MS of selected targets from control and HD cells. Representative graphs showing overlay of indicated transcripts for ribosome occupancy (RPF/mRNA) (n = 3 experiments) extracted from UCSC Genome Browser and corresponding TMT-MS ratio of fold change (n = 5 experiments) derived from Scaffold for Cavin-1 (*A*), Ptcd3 (*B*), Bsg (*C*), Ece1 (*D*), Prxl2a (*E*) and Plpp3 (*F*). Error bar represents mean ± SEM ∗∗∗∗*p* < 0.0001, ∗∗*p* < 0.01, ∗*p* < 0.05, one-way ANOVA followed by Dunnett’s multiple comparison test. HD, Huntington disease; TMT-MS, tandem mass tag–based mass spectrometry; UCSC, University of California Santa Cruz.

We also examined the six most prominent proteins, apart from those associated with OXPHOS, that exhibited enrichment in the mitochondrial fraction. These proteins were also associated with mitochondrial activities, as indicated by the MitoCarta3 annotations in the TMT-MS data (Data Sheet 2). Cavin1, a protein that promotes mitochondrial function and oxygen consumption and participates in HD, shows highly increased levels in HD mitochondria (48, 49). We found an enhanced Cavin1 protein level, but no change in ribosome occupancy, in HD-homo cells (Fig. 10*A*). Ptcd3, a small subunit of the mitochondrial ribosome required for efficient translation (50), showed strong diminishment of both ribosome occupancy and protein levels (Fig. 10*B*). Bsg (aka, CD147), which regulates mitochondrial complex I activity (51), showed no significant alteration in ribosome occupancy, but its protein levels were diminished in HD-het and HD-homo cells (Fig. 10*C*).

Endothelin converting enzyme 1 (Ece1), which participates in the proteolytic conversion of endothelin precursors to physiologically active peptides, showed enhanced ribosome occupancy in the HD-het cells, but its protein levels were upregulated in both HD-het and HD-homo cells (Fig. 10*D*). Peroxiredoxin like 2A (prk12a), a protein involved in redox regulation, showed high Ribo-Seq in HD-het cells but a substantial reduction in the protein levels in both HD-het and HD-homo cells (Fig. 10*E*). Phospholipid phosphatase 3 (plpp3), by contrast, showed no alterations in Ribo-Seq, but its protein levels were enhanced in mitochondria isolated from HD-het and HD-homo cells (Fig. 10*F*).

Taken together, these results indicate that the ribosome occupancy of mitochondria-associated transcripts is not directly correlated with protein abundance. This is the first evidence that ribosome occupancy estimated by translational dynamics does not translate into protein-level changes in HD mitochondria.

## Discussion

The inquiry into whether alterations in protein levels are primarily attributed to variations in protein synthesis rate, mRNA abundance, or protein turnover has been a longstanding topic of investigation in biology. In yeast, the current notion is that the proportion of ribosome-bound mRNA molecules, which signifies actively translated species, is a more reliable indicator of protein abundance than mRNA levels in isolation (52, 53, 54). Here, we integrated ribosome profiling and mass spectrometry-based protein discovery techniques to provide a comprehensive analysis of the proteome in mammalian mitochondria. Our findings offer novel insights into the relationship between ribosome occupancy and the protein levels of mitochondrial proteins in HD cells.

Multiple aspects of mitochondrial dysfunction are linked to numerous brain diseases, particularly neurodegenerative diseases (55, 56, 57, 58, 59, 60, 61, 62, 63, 64, 65). Perturbed mitochondrial functions and disrupted protein synthesis have been reported in HD (3, 16, 66, 67, 68, 69, 70, 71, 72, 73, 74), but a detailed molecular and biochemical understanding is lacking. This report presents compelling evidence for the existence of a distinct dichotomy in the expression patterns of proteins linked to mitochondria. We found defects in translational regulation involving the enhanced loading of ribosomes onto mitochondrially encoded mRNAs that control mitochondrial energy production, cell signaling, and cell death functions (75, 76). Notably, this regulation was absent from the most nuclear-encoded mitochondrial mRNAs, revealing an intriguing mechanistic dichotomy in the translational process operating between the cytoplasm and the mitochondria in the HD context. This dichotomy has the potential to contribute to the observed decrease in mRNA translation in HD mitochondria (Fig. 1).

An enormous pressure appears to exist for mitochondria-encoded transcripts to increase translational efficiency; however, for unknown reasons, this pressure is not translated into protein production. As reported here, there is a general decrease in protein production in HD mitochondria and mitochondrial OXPHOS transcripts. The reason for this misregulated translation efficiency in HD is unclear. One possibility is that the ribosomal proteins that translate mitochondria-encoded OXPHOS transcripts are present at suboptimal levels. Consistent with this notion, of the 38 mitochondrial ribosomal proteins found in our TMT-MS data, 11 were significantly downregulated in HD cells. However, whether this downregulation is the cause or a consequence of enhanced ribosome occupancy is unclear because the stalled ribosomes and mRNA may be subject to no-go degradation (77), resulting in diminished mitochondrial ribosomal protein levels.

**Fig. 11:**
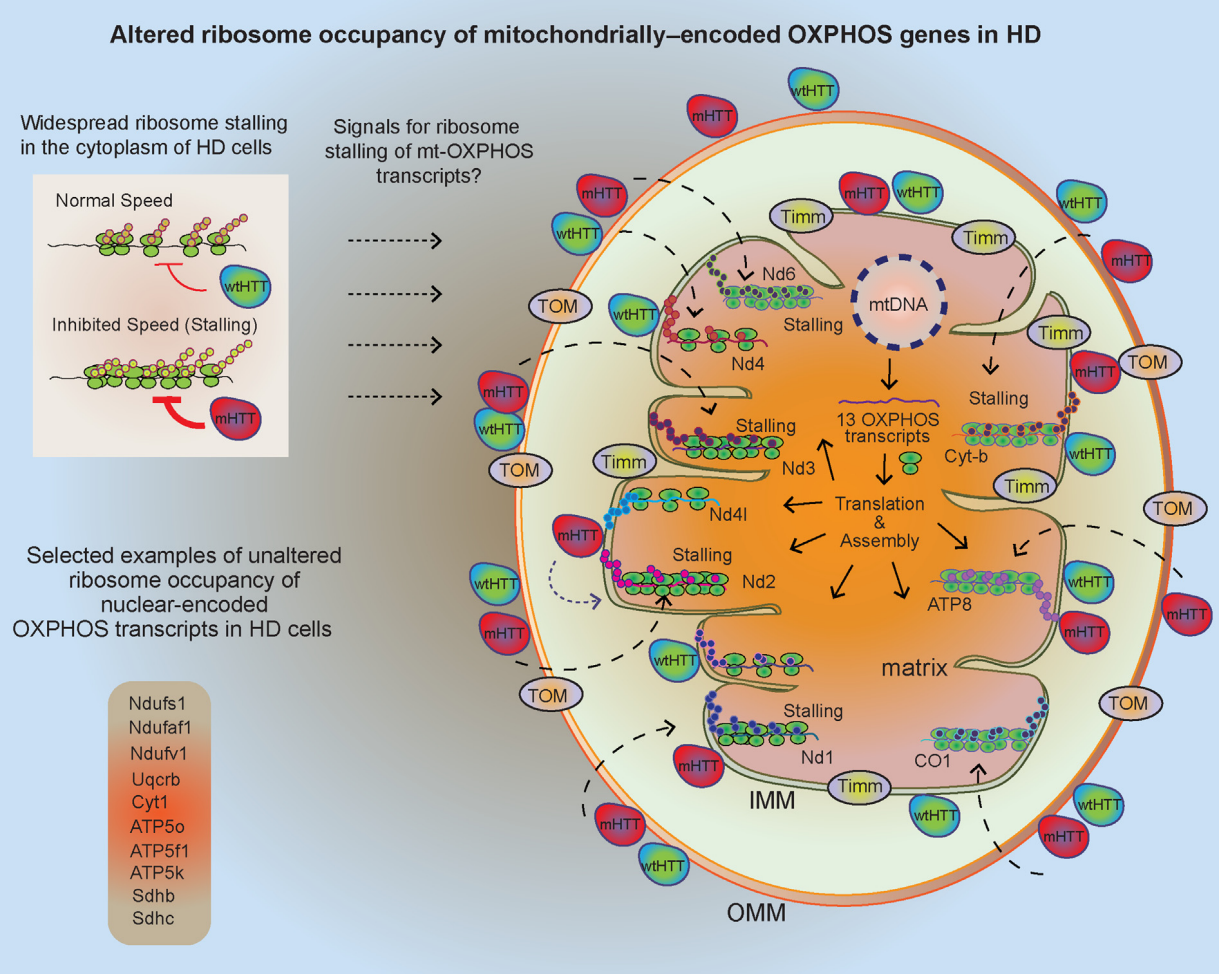
**Model for mHTT-mediated ribosome stalling of mitochondriallyencoded OXPHOS genes.** We reported widespread ribosome stalling in HD cells (35). Our model has predicted that HTT hinders the movement of ribosomes, while mHTT acquires this capability and further obstructs ribosome mobility, leading to the inhibition of protein synthesis. Here, we report an intriguing observation regarding the ribosome occupancy of OXPHOS mRNA transcripts in HD cells. Almost all mitochondrially encoded OXPHOS transcripts, but not the nuclear-encoded OXPHOS transcripts, show a higher ribosome occupancy in HD cells compared to control cells. We hypothesize that the signaling mechanisms by which HTT or mHTT induce ribosome loading of mt OXPHOS transcripts may originate from either the cytoplasm or the inner or outer mitochondrial membrane. The underlying molecular process and its role in HD onset and progression remain unknown. HD, Huntington's disease; HTT, huntingtin; mHTT, mutant huntingtin; OXPHOS, oxidative phosphorylation.

Previous studies have indicated that both wtHTT and mHTT are localized in the mitochondria, but whether they reside in the mitochondrial intermembrane space or in the outer membrane remains controversial (18, 23, 26, 27, 78, 79). Our TMT-MS experiment revealed HTT interacting protein-1, but not the HTT protein itself, in the mitochondrial fractions. Even if this mitochondrial localization is transient, it raises the intriguing possibility that mHTT/wtHTT can induce cellular signaling from the inner or outer membrane surface to regulate ribosome occupancy and cotranslational events involving mitochondria encoded OXPHOS mRNA (Fig. 11). Alternatively, wtHTT/mHTT fragments might directly bind to the polysomes and ribosomal proteins in the cytoplasm, thereby impeding the speed of ribosome translocation and protein synthesis (35).

As most (>95%) of the mitochondrial proteins are produced in the nucleus, one possibility is that HTT/mHTT may activate signals in the nucleus and cytoplasm, and these then communicate messages to the mitochondrion for its own mRNA translation (Fig. 11). Recent studies in yeast have shown that cytosolic translational regulators control mitochondrial OXPHOS mRNA translation (80, 81). In particular, some evidence indicate that a nuclear-encoded protein involved in OXPHOS might impede the assembly process of the nascent chain complex of mitochondrial OXPHOS proteins (82). These discoveries suggest the existence of a functional and physical connection involved in the assembly of mitochondrial proteins. Whether or how such mechanisms impacts HD remains to be discovered (Fig. 11). Identifying the molecular nature of the cellular signals and ribosome stalling mechanisms in HD cells will provide new insights that could help curtail mitochondrial translation defects for a potential therapeutic impact on HD.

One important observation from this study is the differential influence of ribosome occupancy on mitochondrially coded mRNA by one copy of mHTT (HD-het) *versus* two copies of mHTT (HD-homo). When compared to HD-homo cells, the HD-het cells display a significantly higher ribosome occupancy on the mt-OXPHOS genes, with a minority of nu-coded OXPHOS transcripts exhibiting diminished occupancy (Fig. 3, Fig. 4, Fig. 5). This observation suggests that the combined presence of wtHTT and mHTT may lead to the emergence of an additive risk for translation regulatory phenotypes that readapt to ongoing HD-related cellular demands. In addition, as wtHTT plays a key role as a negative regulator of ribosome translocation (83), the loss of normal HTT in HD-het may lead to enhanced loading of ribosomes on mitochondrial OXPHOS transcripts. Alternatively, mHTT may exert a gain of function, so that the presence of two copies of mHTT in HD-homo cells further enhances the degree of ribosome occupancy in mitochondrial OXPHOS transcripts, leading to the collision of ribosomes and eventual no-go degradation of the transcripts. This notion is supported by the significantly reduced amounts of mitochondrial mRNA transcripts in the HD-homo *versus* the HD-het or control cells (Fig. 3, Fig. 4, Fig. 5). This type of process may also be responsible for the much greater diminishment of protein synthesis in HD-homo cells than in HD-het cells (Fig. 1, *A*–*C*). Future studies should investigate the poly-Q–dependent mechanisms of HTT that might induce a decay of ribosome-stalled mitochondrial transcripts and how this could lead to diminished protein synthesis in HD.

A previous study using two-dimensional difference gel electrophoresis indicated reduced levels of nuclear-encoded matrix mitochondrial proteins in HD-homo cells (27). Consistent with this previous report, we also observed a significant reduction in the Idh3a, Slc25a24, and Pck2 protein levels, while Clpp and Me2 showed a decreasing trend that did not reach statistical significance. Thus, independent studies have validated the occurrence of defective mitochondrial proteomes in HD. Nevertheless, our high-throughput methodology has now further advanced the characterization of mitochondrial transcript-specific changes to uncover dynamic translational modifications in HD. For simplicity, the altered transcripts could be grouped into two broad categories comparing control *versus* HD cells: (1) transcripts that had high ribosome occupancy but a diminished or similar protein content (*e.g.*, mt-nd1 and prkl12a) and (2) transcripts that had similar ribosome occupancy but diminished or higher protein levels (*e.g.*, Cox7a2I, Cavin1, and plpp3).

Taken together, our study findings demonstrate an unexpected dichotomy of ribosome occupancy and proteomes among nuclear and mitochondrially coded OXPHOS transcripts due to mHTT. Defining the underlying mechanisms that create this dichotomy of OXPHOS mRNA translation and proteome changes, and determining their influence on mitochondrial structure and function, will provide a new understanding of HD pathogenesis and identify new therapeutic targets.

## Data Availability

All the data are available in the main text or the Supplementary materials.

## Supplemental Data

This article contains supplemental data.

## Conflict of interest

Authors declare no competing interests.

## Supplemental Data

Data Sheet 1

Data Sheet 2

Data Sheet 3
